# Effects of Working Memory Span on Processing of Lexical Associations and Congruence in Spoken Discourse

**DOI:** 10.3389/fpsyg.2013.00060

**Published:** 2013-02-13

**Authors:** Megan A. Boudewyn, Debra L. Long, Tamara Y. Swaab

**Affiliations:** ^1^Department of Psychology and Center for Mind and Brain, University of CaliforniaDavis, CA, USA

**Keywords:** N400, lexical association, discourse congruence, individual differences, working memory capacity

## Abstract

The goal of this study was to determine whether variability in working memory (WM) capacity and cognitive control affects the processing of global discourse congruence and local associations among words when participants listened to short discourse passages. The final, critical word of each passage was either associated or unassociated with a preceding prime word (e.g., “He was not prepared for the fame and *fortune/praise*”). These critical words were also either congruent or incongruent with respect to the preceding discourse context [e.g., a context in which a prestigious prize was won (congruent) or in which the protagonist had been arrested (incongruent)]. We used multiple regression to assess the unique contribution of suppression ability (our measure of cognitive control) and WM capacity on the amplitude of individual N400 effects of congruence and association. Our measure of suppression ability did not predict the size of the N400 effects of association or congruence. However, as expected, the results showed that high WM capacity individuals were less sensitive to the presence of lexical associations (showed smaller N400 association effects). Furthermore, differences in WM capacity were related to differences in the topographic distribution of the N400 effects of discourse congruence. The topographic differences in the global congruence effects indicate differences in the underlying neural generators of the N400 effects, as a function of WM. This suggests additional, or at a minimum, distinct, processing on the part of higher capacity individuals when tasked with integrating incoming words into the developing discourse representation.

## Introduction

Discourse comprehension requires language users to integrate information from multiple levels of meaning, and individuals vary considerably in their use of semantic and syntactic information during sentence and discourse processing. Behavioral studies of individual differences in discourse comprehension have implicated a variety of factors, such as word-decoding ability, suppression ability, working memory (WM) capacity, print exposure, and background knowledge, as contributors to reading skill differences (see Long et al., 2006, for a review). Event-related potential (ERP) studies of individual differences in comprehension are less common, with most focusing on the comprehension of written sentences. Few ERP studies have investigated individual variability during online listening comprehension, but recent work has implicated WM capacity as contributing to variability in processing thematic relations in spoken sentences (Nakano et al., 2010), and controlled suppression ability as contributing to individual differences in processing word-level meaning relations in spoken sentences (Boudewyn et al., 2012b).

Previous studies suggest that WM and cognitive control capacity contribute to individual differences in the ability to construct a globally coherent discourse representation, and also in the degree to which comprehenders are influenced by meaning relations among words (e.g., Gernsbacher, 1990; Gernsbacher and Faust, 1991; Just and Carpenter, 1992; Cantor and Engle, 1993; Gernsbacher, 1997; Van Petten et al., 1997; Vos et al., 2002; Vos and Friederici, 2003; Bornkessel et al., 2004; Nakano et al., 2010; Boudewyn et al., 2012b). The current study, for the first time, combines electrophysiological measures of language processing with measures of cognitive control and WM capacity in order to examine if and how individual variability in these measures affects processing of global discourse-level congruence and local word-level meaning relations. In the following sections, we first discuss studies that have examined the role of lexical associations and/or message-level congruence during the comprehension of incoming words in context. We then review evidence suggesting that individual variation in WM capacity and cognitive control ability may influence the processing of discourse-level meaning and meaning relations among words in discourse context. Finally we turn to specific predictions for this experiment.

### Discourse congruence and lexical associations as levels of meaning in context

Spoken discourse comprehension is a complex dynamic process that requires the rapid translation of acoustic events into meaningful speech and the integration of multiple sources of linguistic and contextual information with the incoming speech signal. Many studies have shown that information from previous context and from background knowledge can be brought to bear quickly in order to interpret and integrate incoming words (see van Berkum, 2009; Swaab et al., 2011 for reviews). Specifically, the processing of incoming words can be facilitated by multiple factors, including (but not limited to) the presence of associatively related prime words in the preceding context (e.g., Van Petten, 1993; Hoeks et al., 2004; Coulson et al., 2005; Camblin et al., 2007; Boudewyn et al., 2012a,b), the previous sentence or discourse context when it is coherent, or predictive of upcoming words (e.g., Federmeier and Kutas, 1999; van Berkum et al., 1999, 2003; Camblin et al., 2007; Diaz and Swaab, 2007; Boudewyn et al., 2012a), and general world knowledge (e.g., Hagoort et al., 2004; van Berkum et al., 2008). As argued by Van Berkum in his “multiple-cause resource-intensified memory retrieval” hypothesis, many types of information contribute to the “interpretive background” of each incoming word in context, all of which can facilitate the retrieval of the meaning(s) of that word (van Berkum, 2009). In ERP studies, facilitation of the processing of incoming words by supportive or consistent interpretive background results in a reduction of the N400 waveform, a negative-deflecting ERP that is sensitive to semantic aspects of input (see Swaab et al., 2011 for a review).

Although many contextual variables impact the processing of incoming input, these factors can vary in terms of their relative influence on processing. Although word-level associations have a significant impact on processing when presented in pairs or lists (e.g., Williams, 1988; Marslen-Wilson and Zwitserlood, 1989), they have a relatively weak influence on processing when embedded in a sentence or discourse context, and are not consistently observed. As noted above, several studies have found lexical association effects for words in context (Van Petten, 1993; Hoeks et al., 2004; Coulson et al., 2005; Camblin et al., 2007; Boudewyn et al., 2012a,b), but other studies have found association effects only under certain conditions, such as when primes and targets are embedded in the same clause (Carroll and Slowiaczek, 1986), when primes and targets are congruent with the sentence-level meaning (Morris, 1994), when primes are in linguistic focus (Morris and Folk, 1998), or when targets appear in incongruent (Coulson et al., 2005) or low-constraint (Hoeks et al., 2004) sentences.

In contrast, evidence from recent ERP studies has shown that discourse-level meaning has a powerful and early influence on the processing of upcoming words, even though some traditional accounts of discourse comprehension have suggested that while the activation of individuals concepts is fast and automatic, integrated representations of discourse context must be retrieved from memory in order to be brought to bear on information currently being processed (Kintsch, 1988; Ericsson and Kintsch, 1995). However, N400 amplitude differences between words that fit well with the larger message compared to words that are less congruent, or anomalous, are found without delay (Federmeier and Kutas, 1999; van Berkum et al., 1999, 2003; Camblin et al., 2007; Boudewyn et al., 2012b). In our studies, we have directly manipulated discourse congruence and lexical association within the same paradigm, and have found congruence effects to be more robust and long-lasting compared to association effects, both during reading and auditory comprehension (Camblin et al., 2007; Boudewyn et al., 2012b).

Although the results of these studies suggest that word-level association has a weaker influence than discourse congruence on processing of incoming words, the relative influence of these factors can vary depending on the particular context. For example, readers sometimes fail to detect semantic anomalies within discourse contexts such as “victim” in the following passage: “Child abuse cases are being reported much more frequently these days. In a recent trial, a 10-year sentence was given to the victim, but this was subsequently appealed” (Sanford et al., 2010). In this example, the passage is fully congruent up until the critical word “victim,” and the failure of many readers to detect this break with the message of the discourse context may be interpreted as an instance in which readers over-rely on the associative relations among words in the context (e.g., “victim” is strongly associated with, and possibly activated by, words in the context). These results suggest that the relative influence of discourse-level meaning and word-level meaning on processing is determined by the particular context being processed.

As previously discussed, language processing is also influenced by individual differences characteristics, and in the current study, we test the hypothesis that the relative influence of discourse-level meaning and word-level meaning on the processing of incoming words is influenced by individual differences in WM capacity and cognitive control ability. These individual differences characteristics were selected for two reasons. First, WM capacity and cognitive control are theoretically important during language comprehension: the maintenance of discourse context places demands on WM resources, and control mechanisms (e.g., suppression ability) are likely involved in the inhibition of context-irrelevant information activated by language context. Second, individual differences in both WM capacity and cognitive control ability have been implicated by previous research as being related to differences in spoken language comprehension (e.g., Nakano et al., 2010; Boudewyn et al., 2012b). Thus, WM capacity and cognitive control ability may be particularly influential in determining sensitivity to message-level meaning and lexical association during discourse processing, as larger, multi-sentence discourse passages place great demands on WM maintenance and on controlled integration of context-relevant information.

### Working memory capacity and language processing

Working memory capacity has long been implicated in theories of individual differences in language processing, and particularly in studies investigating variability in the reading of syntactically complex sentences (Just and Carpenter, 1992; Vos et al., 2002; Vos and Friederici, 2003; Bornkessel et al., 2004). There are three major accounts of the role of WM during language comprehension. The first two accounts focus on the limited-capacity of WM resource(s) (e.g., Just and Carpenter, 1992; Waters and Caplan, 1996). According to the Capacity Theory (Just and Carpenter, 1992), language processing and context maintenance draw upon a single limited-capacity WM resource pool (of activation). Variability in the amount of activation available, or capacity, produces individual differences in sentence processing. This is especially apparent for complex syntactic structures that are resource-demanding, and leave few available resources for the maintenance of non-syntactic information such as the meaning of individual words or the sentence context as whole. Similarly, Mason and Just (2004) suggest that the processing of discourse context requires WM resources to maintain contextually relevant information, so that comprehenders can create anaphoric references and inferences. Therefore, low WM capacity individuals are expected to be limited in their ability to maintain contextual information during the processing of complex sentences and discourse context. In contrast, the Separate Sentence Interpretation Resource (SSIR) theory (Waters and Caplan, 1996) proposes that there are two separate WM resource pools that may be tapped during sentence processing. One resource pool underlies initial syntactic structure-building, and does not vary across individuals. However, individuals vary in the capacity of the second resource, which is more general and comes into play during controlled, verbally mediated tasks such as a conscious search of memory or logical reasoning (Waters and Caplan, 1996). Even though Waters and Caplan (1996) made no explicit predictions with respect to this second resource relative to discourse processing, it is reasonable to assume that discourse processing would tap into this resource as well, and that low capacity comprehenders would have greater difficulty in maintaining context-relevant information. Thus, even though the first two accounts of the influence of WM capacity on language processing make different predictions with respect to the processing of syntactic information, they would likely make similar predictions regarding the influence of WM capacity on discourse processing.

The third account regarding the role of WM in language comprehension attributes variability to individual differences in processing efficiency, arising as a function of language experience. This alternative proposal to limited-capacity models has been put forward by MacDonald and Christiansen (2002), who argue that “capacity” is a property of the efficiency and experience of the processing system as a whole, rather than a limited resource pool. According to this account, variability in sentence processing is a result of individual differences in reading skill and experience (MacDonald and Christiansen, 2002). Complex syntactic structures cause processing delays or difficulties, not because they are resource-demanding, but because they are less frequently encountered than simpler structures, which are more common. Skilled readers have more experience than less skilled readers with complex syntactic structures. Therefore, facilitated comprehension of difficult structures can be explained as a result of experience, rather than of “high-capacity.” Indeed, there is evidence indicating that increased exposure to complex syntactic structures can modify processing of these structures in “low-span” subjects (MacDonald et al., 1994; Long and Prat, 2002).

Whether individual variability on WM tasks and during sentence comprehension is best attributed to differences in a limited-capacity resource pool(s) or to differences in language experience, the fact that individuals vary in their ability to construct and maintain language context has important implications for our understanding of language comprehension. As noted above, most studies of the relation between WM capacity and language processing have focused on the reading of syntactically ambiguous or complex sentences (Just and Carpenter, 1992; Vos et al., 2002; Vos and Friederici, 2003; Bornkessel et al., 2004). Here we will review studies that are particularly relevant to the current study because they have investigated the relation between WM capacity and the use of meaning information during sentence or discourse comprehension.

First, there is some evidence to suggest that high WM capacity individuals integrate meaning across sentences in discourse context more quickly and effectively than low WM capacity individuals (Cantor and Engle, 1993, Experiment 2). In Cantor and Engle, WM span was measured in the following way: Participants were presented with mathematical operation-word pairs, in groups ranging from two to seven [e.g., (6/2) – 2 = 1; DOG]. Following presentation and completion of all operation-word pairs, participants were asked to recall as many words from the previous group as possible. The sum of correctly recalled words (from groups where recall was perfect) was taken as the WM span measure. Participants then read a series of passages that were either three or six sentences long, and featured different subject nouns (e.g., the teacher). Participants were asked to recall the sentences associated with each subject (e.g., all sentences about the teacher), and repeated the study-recall cycles until they were able to accurately recall all items in a particular passage on three consecutive trials. Following this learning phase, a verification memory test was given to the participants, in which they were asked to identify whether or not experimental and foil sentences had been present in the study phase of the experiment. In contrast to low WM capacity individuals, high WM capacity participants required fewer study cycles in order to accurately recall the passages, and were faster to recognize sentences that had appeared in longer passages compared to the shorter three-sentence passages (Cantor and Engle, 1993). This suggests that high-capacity individuals utilized the additional information in the longer passages to construct richer, more durable discourse representations. In contrast, low WM capacity individuals exhibited the opposite pattern, and were faster to recognize sentences that had appeared in the shorter three-sentence passages. This pattern of results suggests that the high WM span group integrated the individual sentences of the longer passages into a coherent mental model more quickly and successfully than the low WM span group (Cantor and Engle, 1993). Therefore, higher capacity individuals may be better able than lower-capacity individuals to maintain and integrate across multiple sentences in order to construct a unified discourse representation during comprehension.

Other studies have investigated the relation between WM capacity and the processing of meaning information during comprehension using ERPs. One study has linked WM capacity with sensitivity to word-level animacy information in spoken sentences (Nakano et al., 2010). Specifically, Nakano et al. (2010) found that ERP signatures in sentences that violate the thematic constraints of verbs (e.g., *biting* in “The box was biting the mailman”) compared to word-knowledge-violated or non-anomalous verbs (e.g., *biting* in “The poet/dog is biting the mailman”) varied as a function of WM span: Thematic violations elicited a P600 effect in high-span individuals, but an N400 effect in low-span individuals (Nakano et al., 2010). N400 effects are typically produced by varying semantic fit across conditions, whereas P600 effects are linked to syntactic manipulations or conflict at the semantics-syntax interface (see Swaab et al., 2011 for a review). Therefore, the pattern of results in this study suggests that high-span, but not low-span, participants were able to quickly make use of animacy information from the first noun in the sentence (i.e., that an inanimate noun such as *box* is not a suitable subject for a verb like *biting*) in order to begin thematic role assignment, leading to a P600 effect at the verb when the interpretation requires revision [i.e., that the syntax dictates *box* is actually the (anomalous) subject of the sentence]. In another ERP study, participants read sentences that contained associated or unassociated word pairs, and that were either fully congruent or anomalous (Van Petten et al., 1997). Only high WM participants distinguished between congruent and anomalous sentences when no word-level associations were present, whereas all participants showed N400 effects of word-level association, regardless of WM capacity, and of sentence congruence, when sentences contained associations (Van Petten et al., 1997). This pattern of results suggests that low WM capacity individuals are less sensitive than high-capacity participants to sentence-level meaning.

### Cognitive control ability

Cognitive control ability may also play a crucial role in discourse processing (Gernsbacher, 1990, 1997). It is important to note that cognitive control is somewhat of a umbrella term, often used to refer to any kind of controlled process, from the suppression of irrelevant input through controlled maintenance of information. In the current study, we focused on one specific aspect of controlled processing: the ability to suppress or inhibit irrelevant information. This is particularly important given that suppression ability has been proposed to be related to individual variability in discourse comprehension according to a prominent theory of discourse comprehension, the Structure-Building Framework (Gernsbacher, 1990, 1997; Gernsbacher and Faust, 1991). This framework emphasizes the cognitive processes of mapping, which involves incorporating incoming input into the current mental representation, and shifting, which involves constructing a new mental representation with the incoming input. Mapping occurs when incoming input fits well with the current mental representation, whereas shifting occurs when the input is less coherent (Gernsbacher, 1990). Successful construction of meaning representations requires the enhancement of context-relevant information, and the suppression of context-irrelevant information that may be activated by incoming words and phrases. For example, the “playing card” meaning of the word *spade* would be activated, even though it is context-irrelevant, in a sentence involving the “garden tool” meaning of *spade*. Ineffective suppression of irrelevant information may lead to excessive shifting, and therefore to the creation of unnecessary sub-structures and disorganized discourse representations (Gernsbacher, 1990, 1997; Gernsbacher and Faust, 1991).

As noted in the “playing card” vs. “garden tool” example, words that are related in meaning to the context-inappropriate sense of ambiguous words (e.g., *ace* to *spade* in “He dug with the spade”) represent one source of irrelevant information that is activated during comprehension. Gernsbacher and colleagues presented sentences containing ambiguous words such as “spade” in the example above, followed by probe words that were, in some cases, related to the context-inappropriate meaning of the word (*ace*), and asked to respond as to whether or not the probe word was related to the sentence. Less skilled readers were found to be slower than skilled readers at rejecting test words when they were context-inappropriate (Gernsbacher and Faust, 1991; Experiment 4). This result provides evidence that skilled readers more effectively suppress irrelevant information during comprehension, and that low skilled readers are influenced to a greater extent by local associations among word meanings.

Further evidence for the role of cognitive control in language comprehension comes from several neuroimaging studies that have found regions associated with domain-general cognitive control functions, such as the dorso-lateral prefrontal cortex (DLPFC) to be active under difficult sentence processing conditions, such as complex or ambiguous sentence structures (January et al., 2009; for a review, see Novick et al., 2005, 2010). The DLPFC has also been implicated during the maintenance of simple sentence contexts, with increased activation during maintenance linked to context-appropriate responding in a word-completion task (Kerns et al., 2004). Therefore, domain-general control, mediated by the DLFPC, may be recruited even during simple sentence comprehension in order maintain context information and guide processing.

### Predictions for the current study

The goal of this study was to use ERPs to investigate individual variability in sensitivity to discourse-level congruence and lexical association during spoken discourse comprehension. Discourse congruence was manipulated by constructing three-sentence story contexts in which the final, critical word was consistent with the meaning of the preceding context (discourse congruent) or not (discourse incongruent). For example, the critical word “fortune” in the sentence “He was not prepared for the fame and *fortune*” would be discourse congruent following a context in which a prestigious prize is won, but discourse incongruent following a context in which the protagonist had been arrested. In addition, the final critical words were either associated to a preceding prime word (“…fame and *fortune*) or unassociated (“…fame and *praise*). We tested whether differences in WM capacity and cognitive control contribute to differences in the use of global discourse context and local meaning relations, using multiple regression to assess the unique contribution of these measures on the amplitude of the N400 congruence and association effects. This study is one of only a handful of ERP studies that have applied a multiple regression approach to investigate individual variability in ERP effects (see Dambacher et al., 2006; Laszlo and Federmeier, 2011; Boudewyn et al., 2012b, for other examples), and is the first to directly assess relations among measures of WM and cognitive control, and the real-time comprehension of spoken discourse. This is an important goal, as previous studies have focused primarily on the reading of single sentences. Although sentence processing studies are informative, and have suggested a link between WM and the ability to maintain language context (e.g., Van Petten et al., 1997), as well as between control (suppression ability), and the suppression of irrelevant information during comprehension (Gernsbacher, 1990, 1997; Gernsbacher and Faust, 1991), there is good reason to think that control and WM processes play a larger role during the processing of discourse context. Discourse comprehension places greater demands on language users than sentence comprehension, in that language users must integrate across sentences in order to construct a message-level representation of the meaning of a passage. In addition, the current representation must be maintained in WM in order for incoming input to be integrated. Finally, discourse context contains information at multiple levels, as mentioned above, which may activate context-irrelevant words or concepts, placing demands on suppression ability.

It is important to note that cognitive control and WM may not be separable constructs, particularly given that performance on WM tasks has been linked to performance on cognitive control tasks (see Engle, 2002 for a review). Therefore, in this study, we have adopted the approach of (1) specifying which aspects of control (suppression ability) and WM (maintenance) we wish to investigate, and (2) adopting a multiple regression approach in order to account for the unique contribution of each measure in predicting individual ERP effects related to language processing. Importantly, the tasks employed in the current study were designed to tap into different aspects of controlled processing. Our control measure, the Stroop task, is primarily a measure of suppression ability, as participants are asked to respond to the font color of printed color words (e.g., red for the words *blue/red* when printed in red). Trials involving a mismatch between the color term and the font color (e.g., *blue* in red) require that participants overcome conflict and suppress the meaning of the word in order to respond appropriately. In contrast, our WM task requires that participants listen to sets of sentences, evaluate whether, or not each one is a true statement, and keep the last word of each sentence in a set in mind to be recalled after the set ends. In short, it is a dual-task paradigm, involving memory but also the controlled allocation of attentional resources and the maintenance of information over time. Therefore, although there is likely to be some overlap between control and WM, the control and WM tasks used in this study tap into different aspects of controlled processing.

Given that previous work has linked WM capacity with sensitivity to sentence- (Van Petten et al., 1997; Nakano et al., 2010) and discourse context (Cantor and Engle, 1993), we predicted that WM span would significantly predict ERP effects of congruence. Specifically, high WM span was expected to predict larger N400 effects of congruence (larger N400 deflection to incongruent target words than congruent target words). Likewise, if individuals with lower WM spans are less able to maintain and use language context, and are therefore less sensitive to discourse congruence, then lower WM span individuals may have a smaller portion of the preceding context active and available in WM than higher WM span individuals. Critically, the associated prime words appear in the local context immediately preceding the target words, leading the local primes to occupy a larger, more prominent position in the portion of context that is active for lower WM span individuals than for higher WM span individuals. If this is the case, then lower WM span individuals may prove to be more sensitive to the local word-level context (associations; larger N400 deflection to unassociated target words than associated target words). In other words, local word-level context may increase in salience as a function of WM span, in that word-level context may be more influential for lower-span individuals who are less able to maintain the larger, discourse-level context. In short, N400 effects of discourse congruence are expected to increase with higher WM span, whereas N400 effects of lexical association are expected to decrease as a function of higher WM span.

In contrast, we predicted that cognitive control (suppression) ability, as measured by the Stroop task, would predict sensitivity to the presence of local lexical associations. As noted above, suppression of irrelevant information is an important aspect of language comprehension, and skilled readers have been shown to be more effective at suppressing context-irrelevant lexical associations during sentence comprehension (Gernsbacher et al., 1990). Given the task of listening to connected discourse for comprehension, the presence of lexically associated words has been shown to be a weak source of information compared to discourse-level context, resulting in smaller N400 effects (Camblin et al., 2007; Boudewyn et al., 2012a). In this case, it may be important to suppress lexical associates during discourse comprehension. Indeed, in a previous study, we found that high control participants showed smaller N400 effects of lexical association for words in sentences (Boudewyn et al., 2012a). Following from this, in the current study, we predicted that increased suppression ability would be associated with smaller effects of association (larger N400 deflection to unassociated target words than associated target words), and potentially with larger effects of congruence (larger N400 deflection to incongruent target words than congruent target words).

## Materials and Methods

### Participants

Twenty-six undergraduates (17 female) from the University of California, Davis gave informed consent before participating in the study. All were right-handed, native speakers of English, with no reported problems with hearing or reading or neurological/psychiatric disorders. They were compensated with either course credit or at a rate of 10 dollars per hour. The mean age of participants was 20.38 (range: 18–29; SD: 2.45).

### Methods: ERP session

#### Stimuli

The stimuli included 72 experimental story sets, in which discourse congruence (congruent or incongruent) and lexical association (associated or unassociated) was orthogonally varied to produce four conditions (see Table 1): congruent-associated; congruent-unassociated; incongruent associated; incongruent unassociated. These stimuli were adapted from those used in Camblin et al. (2007) and are the same as those used in Boudewyn et al. (2012a). The first two sentences of each passage established a discourse context and the third (final) sentence contained the associated or unassociated target word (see Table 2). Target words were either congruent or incongruent with the discourse context. All except 22 of the prime and target pairs were separated by one intervening word; the remaining stimuli had either two intervening words (8), or three (14). Target words were always congruent with the meaning of the final sentence. That is, target words made sense in context if the sentence were to be heard in isolation.

**Table 1:** **Example stimulus sets showing each of the four conditions described in the text**.

Condition	Context	Target sentence
Associated congruent	Rick was unaware that his sister had submitted his poem in the prestigious contest. He was shocked when he won the award and the hefty cash prize	He was not prepared for the *fame* and FORTUNE
Unassociated congruent	Rick was unaware that his sister had submitted his poem in the prestigious contest. He was shocked when he won the award and the hefty cash prize	He was not prepared for the *fame* and PRAISE
Associated incongruent	Rick was mortified when the videotape of his arrest was shown on the news. After the news show aired, he was ridiculed by the entire neighborhood	He was not prepared for the *fame* and FORTUNE
Unassociated incongruent	Rick was mortified when the videotape of his arrest was shown on the news. After the news show aired, he was ridiculed by the entire neighborhood	He was not prepared for the *fame* and PRAISE

Associated congruent	Although he tried very hard, Ben’s cooking skills were pathetic at best. His latest attempt at making marinara sauce was particularly bland and unappetizing	Luckily Ben had picked up some *salt* and PEPPER
Unassociated congruent	Although he tried very hard, Ben’s cooking skills were pathetic at best. His latest attempt at making marinara sauce was particularly bland and unappetizing	Luckily Ben had picked up some *salt* and BASIL
Associated incongruent	Todd slipped on a large patch of ice near his front step. He wanted to be sure the ice melted before anyone else took a fall	Luckily Todd had picked up some *salt* and PEPPER
Unassociated incongruent	Todd slipped on a large patch of ice near his front step. He wanted to be sure the ice melted before anyone else took a fall	Luckily Todd had picked up some *salt* and BASIL

*For clarification, the primes are shown in italics and target words are capitalized; during the experiment these words were not specifically emphasized*.

**Table 2:** **Results of discourse congruence and lexical association from repeated measures ANOVA, including topographic factors of Anteriority and Hemisphere**.

300–500 ms	*F*	*p*
Congruence	7.56	*
Association	32.99	***
Congruence × association	<1	ns
Congruence × hemisphere	<1	ns
Association × hemisphere	<1	ns
Congruence × association × hemisphere	<1	ns
Congruence × anteriority	<1	ns
Association × anteriority	<1	ns
Congruence × association × anteriority	7.62	*
Congruence × hemisphere × anteriority	2.67	ns
Association × hemisphere × anteriority	<1	ns
Congruence × association × hemisphere × anteriority	3.72	^∧^

Degrees of freedom for all *F* values are 1, 25. ***p < 0.001, **p < 0.01, *p < 0.05, ^∧^*p* < 0.07.

For the association manipulation, associated prime-target word pairs were selected using the Edinburgh Associative Thesaurus (Kiss et al., 1973) as well as association pre-tests conducted as part of a previous study (Camblin et al., 2007). Associated target words had an average association strength of 39.8% (range: 20–90%), while unassociated words pairs had an average association strength of 0.2% (range: 0–4%). For the congruence manipulation, previous pre-testing of the congruence of the stimuli showed that incongruent stories were significantly rated as less congruent than the stories in the congruent conditions on a five-point scale (1 = completely congruent; 5 = completely incongruent). Average congruence ratings were: 1.44 (Congruent/Associated); 1.5 (Congruent/Unassociated); 4.31 (Incongruent/Associated); 4.32 (Incongruent/Unassociated). In order to match across conditions for story coherence and semantic overlap, story sets were matched across conditions using LSA values, which are a measure of similarity of meaning among words, sentences, or multi-sentence stories (Landauer et al., 1998). LSA scores were computed using the “sentence comparison” function (available at http://lsa.colorado.edu/), and the “general reading up to first year college” semantic space (300 factors). LSA values were obtained between sentence 1 and sentence 2 of each passage, and between sentence 2 and sentence 3; the average was computed and served as the LSA score for that item. Values ranged from 0.21 to 0.22 across conditions.

Overall story cloze probability was low (all items <33%); and was below 10% in three of the conditions (Congruent/Unassociated: 7%; Incongruent/Associated: 6%; Incongruent/Unassociated: 3%). In the Congruent/Associated condition the average was 29%. With respect to criteria used in previous ERP and behavioral studies (e.g., Federmeier and Kutas), all of these values constitute low cloze probability. However, in order to confirm that cloze probability did not influence the results, a cloze-matched subset of materials were tested in two previous studies, which showed that cloze probability did not change or contribute to the pattern of results (Camblin et al., 2007; Boudewyn et al., 2012a). The critical target words were matched for frequency across conditions using Francis and Kucera (1982) (Associated = 83.71; Unassociated = 83.9). Target word duration did not differ between conditions (see below).

Story sets were divided into four lists and counterbalanced such that the critical target words and the two sentences preceding the final sentence were not repeated within-subjects. Each list contained 144 experimental stories, 36 in each condition. An additional 40 filler stories were included; 20 were congruent and 20 ended with a word that was anomalous at both the discourse and sentence-level.

Story sets and fillers were spoken by a female speaker, with natural inflection and at a natural speaking rate. The words were digitally recorded using a Schoeps MK2 microphone and Sound Devices USBPre A/D (44,100 Hz, 16 bit). Speech onset and offset of each word was determined by visual inspection of the speech waveform and by listening to the words using speech editing software (Audacity, by Soundforge). Discourse context (first two sentences of the passage) and the critical sentences were recorded separately. The onset and offset of each critical word in all conditions was determined by visual inspection of the speech waveform and by listening to the words using speech editing software (Audacity, by Soundforge). The average duration of the stories was 8951 ms (ranging from 7917 to 9359 ms), and the average duration of the critical words was 568 ms (ranging from 293 to 861 ms). The duration of the critical words did not differ between conditions (*t* < 1). The duration between prime and target was the same for the congruent/incongruent associated and for the congruent/incongruent unassociated conditions because the same final sentence was used for the two associated conditions and the two unassociated conditions. This was accomplished by inserting a 1-s silence between the speech files that contained the first two context sentences and the speech files that contained the final sentences using Presentation software^1^. There were no statistically significant differences in duration of the onset of the prime to the onset of the target between the associated and unassociated conditions (*p* = 0.43). The target sentences were the same in all conditions up to the sentence-final target word.

Comprehension questions did not focus on the prime or the target but rather on the context of the discourse. The same true/false question was asked in the congruent associated/unassociated and incongruent associated/unassociated conditions. Half of the questions required a true response, and half required a false response.

#### Procedure

The ERP session always preceded the behavioral testing session. ERP and behavioral testing sessions were always conducted on consecutive days, with the exception of two participants, who completed the behavioral testing session as part of an unrelated study 4 months prior to their ERP session. Participants were informed at the beginning of the ERP session that the experiment was focused on language comprehension. During the ERP session, participants were seated in a comfortable chair in an electrically shielded, sound-attenuating booth. The stimuli were presented through Beyer dynamic headphones using Presentation software. The discourse trials began with a white fixation cross at the center of the screen, approximately 100 cm in front of the participants. The fixation cross was present from 900 ms before onset of the stimuli and during presentation of the entire passage until the offset of the final word. The fixation cross was then replaced by a visually presented comprehension question about the preceding discourse, after 1000 ms. Subjects were asked to make a true/false response by pressing a yes or no button with the index fingers of each hand. The comprehension question remained on the screen until participants made a response.

Each experimental session began with a practice block consisting of filler passages, after which two of the four counterbalanced lists were presented in random order, each containing both experimental trials and fillers in a pseudorandom order. Each list was divided into four blocks for presentation purposes such that each participant listened to eight blocks of stimuli (two lists). The order of blocks was counterbalanced.

Participants were asked to keep their eyes fixated on the white fixation cross and to refrain from blinking or eye-movements as long as it remained on the screen. This was done in order to minimize movement-related artifacts in the EEG signal. When the fixation cross was replaced by the true/false question, participants were instructed that they could blink and move their eyes freely until they made a response. Condition-specific stimulus codes were sent out at the onset of the critical words and these codes were used for later off-line averaging of the EEG signal.

#### ERP recording and data reduction

EEG was recorded from 29 tin electrodes, mounted in an elastic cap (ElectroCap International). The right mastoid electrode was used as the recording reference (except for electrodes used to measure blinks and eye-movements: one electrode beneath the left eye was referenced to FP1 and two placed at the outer canthi of each eye were referenced to each other). The left mastoid was also recorded for later off-line algebraic re-referencing. The EEG signal was amplified with band pass cutoffs at 0.01 and 30 Hz, and digitized online at a sampling rate of 250 Hz (Neuroscan Synamp I). EEG was digitized continuously along with accompanying stimulus codes used for subsequent averaging. Impedances were kept below 5 kΩ.

Prior to off-line averaging, all single-trial waveforms were screened for amplifier blocking, muscle artifacts, horizontal eye-movements, and blinks over epochs of 900 ms, starting 100 ms before the onset of the critical target words. Average ERPs were computed over artifact-free trials in the related and unrelated conditions. All ERPs were filtered off-line with a Gaussian low-pass filter with a 25 Hz half-amplitude cutoff. Statistical analyses were conducted on the filtered data.

### Methods: Behavioral battery

Before beginning the behavioral battery session, participants were told that the battery was composed of a series of psychological tests designed to assess constructs such as memory, and that some mistakes were to be expected. The instructions for each task were given just prior to task presentation.

#### Listening span

The Listening Span task was adapted from Daneman and Carpenter (1980), and consisted of 25 sets of sentences ranging from two sentences per set to six; there were five sets of each set length. Participants were instructed to listen to all sentences within each set for comprehension, and then to indicate whether each sentence was true or false immediately after hearing the whole sentence. In addition, participants were instructed to remember the final words of each sentence in the set, and were asked to recall them in any order after the whole set was presented. There was a 1500 ms pause in between each sentence during which participants made their true/false response. Presentation of sets was random. Each correct response (correct recall of the final words) was scored as one point, for a maximum of 100 points. This task, which was adapted from its visual counterpart (Reading Span), predicts reading comprehension, and syntactic parsing abilities, particularly when used in conjunction with other tasks (Waters and Caplan, 2003). Further, Nakano et al. (2010) showed that visual and auditory span measures are highly correlated, and are also sensitive predictors of individual differences in spoken language comprehension.

#### Modified Stroop

The Modified Stroop Task was adapted from that used in Van Veen and Carter (2005), and consisted of visual presentation of color words (blue, green, yellow, and red). Words were printed in each of these colors, and participants were instructed to respond to the color of the font and not the word itself; responses were mapped such that a left button press indicated the word was in red or yellow font color, and a right button press indicated that the word was in blue or green font. Trials were either congruent (e.g., “red” in red; 50%), semantically incongruent (SI) in that the font color, and word were not the same but were mapped onto the same finger (e.g., “red” in yellow; 25%), or response incongruent (RI), in that the font color and word were not the same and additionally were not mapped onto the same finger (e.g., “red” in green; 25%). There were 124 total words presented in random order per block, and three experimental blocks. Each word appeared on the screen for 300 ms, separated by a 2700 ms inter-stimulus interval. This version of the Stroop task was designed to separate conflict at the representational level and conflict at the response level by including SI trials in addition to RI and congruent trials. RI trials are assumed to reflect both semantic and response conflict, because they involve a mismatch between (1) the font color and the color being named, and (2) the button response required for the font color and the button response that is required for the color being named. An example of an RI trial is the word “red” printed in green font, when red font has been mapped to one button/finger and green font has been mapped to another. In contrast, SI trials are assumed to reflect only semantic conflict because the button response for both the font color and the color being named is the same. An example of an SI trial is “red” printed in yellow font, when both red and yellow fonts are mapped to the same response button/finger (De Houwer, 2003; Van Veen and Carter, 2005). In a previous study, we showed that this task significantly predicts the size of N400 association effects in spoken sentences (Boudewyn et al., 2012a).

### Results: Behavioral data

#### Comprehension questions

On average, participants responded with 92.7% accuracy (SD: 4.4; range: 81.2–99.3%) on the true/false comprehension questions that followed each story. Accuracy did not differ among experimental conditions, with participants answering 93.5, 93, 92, and 92.5% correctly for the congruent/associated, congruent/unassociated, incongruent/associated, and incongruent/unassociated conditions, respectively.

#### Listening span

Listening Span was calculated as the total number of correctly recalled items (out of a total of 100). The average score was 63.65 (range: 42–79; SD = 10.18).

#### Modified Stroop

Mean RTs were 644.21, 651.82, and 697.75 ms for congruent (C), SI, and RI words, respectively. Planned contrasts revealed no significant difference between the C and SI conditions (*p* = 0.1), but there was a significant difference between the C and RI conditions (*p* = 0.0001). Mean accuracy was 95.7% (C), 95% (SI), and 92.7% (RI); the difference in accuracy between the C and RI conditions was significant (*p* = 0.001). Planned contrasts revealed no significant difference in accuracy between the C and SI conditions (*p* = 0.14), but there was a significant difference between the C and RI conditions (*p* = 0.01). For the purposes of the regression and correlation analyses reported below, a single measure of Stroop Interference was created from the Stroop RT scores. Specifically, for each subject, the average difference between RI and C trials was calculated, following from previous work using this task (Van Veen and Carter, 2005; Boudewyn et al., 2012a).

#### Correlations between behavioral measures

Listening Span performance and Stroop Interference scores were not significantly correlated (*p* = 0.654).

### Results: ERP data

**Figure 1:**
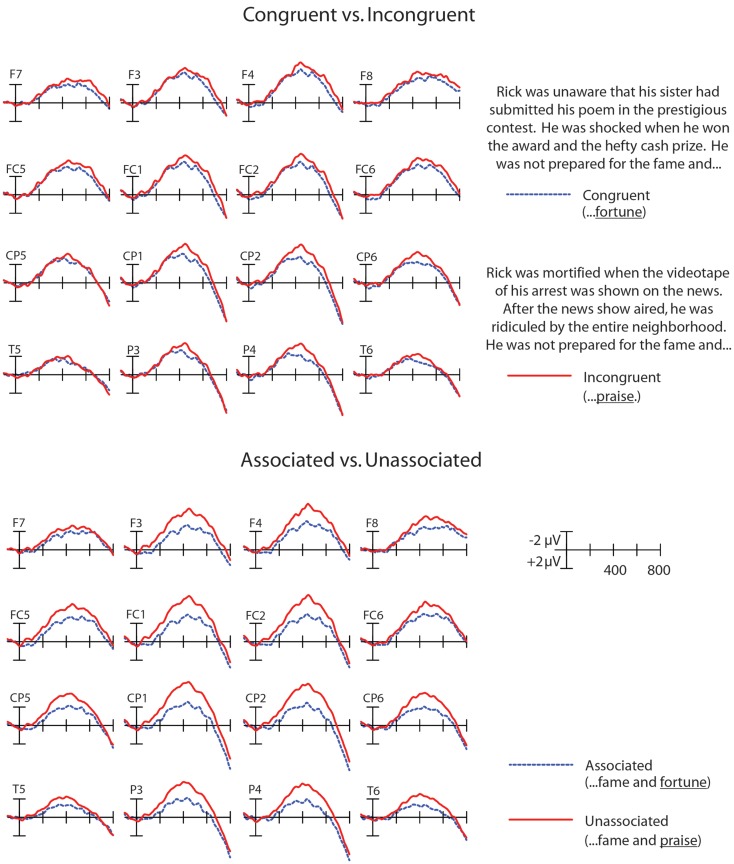
**Main effects of congruence (top) and association (bottom) shown for electrodes in all four quadrants: Left/Frontal (F3, F7, FC1, FC5), Right/Frontal (F4, F8, FC2, FC6), Left/Posterior (CP1, CP5, P3, T5), and Right/Posterior (CP2, CP6, P4, T6)**.

The grand averages across all participants showed effects of discourse congruence and association, such that the N400 waveform to congruent words was reduced compared to incongruent words, and the N400 waveform to associated words was reduced compared to unassociated words (see Figure 1). A repeated measures ANOVA was conducted in the 300–500 ms N400 epoch with the factors Congruence (Congruent, Incongruent), and Association (Associated, Unassociated), and the topographic factors of Anteriority (Anterior, Posterior) and Hemisphere (Left, Right), thereby creating four quadrants of electrodes: Left/Frontal (F3, F7, FC1, FC5). Right/Frontal (F4, F8, FC2, FC6), Left/Posterior (CP1, CP5, P3, T5), and Right/Posterior (CP2, CP6, P4, T6) as within-subjects factors. This configuration was chosen because we were interested in potential differences in the topographic distribution of effects of WM and Control on our manipulations of congruence and association. If participants recruit more WM and/or control processes to maintain contextually relevant information, than these effects could have a more frontal distribution, as frontal ERP effects have often been linked to controlled maintenance (e.g., Kiss et al., 2007). In contrast, if they are heavily influenced by word-level meaning relations among single words, then these effects could have a more typical, posterior N400 distribution (see Swaab et al., 2011, for a review). The Greenhouse–Geisser correction was applied to all *F* tests with more than one degree of freedom in the numerator. These analyses showed a main effect of Congruence [*F*(1,25) = 7.56; *p* < 0.05], a main effect of Association [*F*(1,25) = 32.99; *p* < 0.001]. In addition, there was a significant interaction of Congruence by Association by Anteriority [*F*(1,25) = 7.62; *p* < 0.05], and a marginal interaction of Congruence by Association by Hemisphere by Anteriority [*F*(1,25) = 3.72; *p* = 0.0653]. These results are reported in Table 2.

In order to follow-up on the interactions of Congruence and Association with topographic factors, additional repeated measures ANOVAs were conducted for each electrode quadrant, separately (Factors: Congruence (Congruent, Incongruent), Association (Associated, Unassociated), and Electrode Site (four sites) as within-subjects factors. Significant main effects of congruence were found for the Right/Frontal and Right/Posterior quadrants; the effect of congruence was marginal in the Left/Frontal (*p* = 0.0591) quadrant, and did not reach significance in the Left/Posterior quadrant [but did significantly interact with Electrode (*p* < 0.05) in this quadrant]. In contrast, main effects of Association and interactions of Association by Electrode were found across all four quadrants (*p* < 0.001). In addition, there was a trend toward a Congruence by Association interaction (*p* = 0.088) in the Right/Frontal quadrant. Results for these analyses are reported in Table 3.

**Table 3:** **Results of discourse congruence and lexical association from repeated measures ANOVA, for each quadrant separately**.

300–500 ms	*Df*	*F*	*p*
**LEFT/FRONTAL**			
Congruence	1.25	3.91	^∧^
Association	1.25	27.1	***
Congruence × association	1.25	<1	ns
Congruence × electrode	3.75	<1	ns
Association × electrode	3.75	13.43	***
Congruence × association × electrode	3.75	<1	ns
**RIGHT/FRONTAL**			
Congruence	1.25	4.79	*
Association	1.25	30.71	***
Congruence × association	1.25	3.15	ns
Congruence × electrode	3.75	<1	ns
Association × electrode	3.75	10.09	***
Congruence × association × electrode	3.75	<1	ns
**LEFT/POSTERIOR**			
Congruence	1.25	4.04	^∧^
Association	1.25	25.58	***
Congruence × association	1.25	<1	ns
Congruence × electrode	3.75	5.12	*
Association × electrode	3.75	26.9	***
Congruence × association × electrode	3.75	<1	ns
**RIGHT/POSTERIOR**			
Congruence	1.25	8.35	**
Association	1.25	26.9	***
Congruence × association	1.25	<1	ns
Congruence × electrode	3.75	4.46	*
Association × electrode	3.75	15.48	***
Congruence × association × electrode	3.75	<1	ns

***p < 0.001 **p < 0.01 *p < 0.05 ^∧^*p* < 0.07.

### Results: Regression analysis

#### Dependent measures

We estimated the size of individual N400 effects by calculating the mean amplitude of the effects of Congruence (Incongruent – Congruent) and Association (Unassociated – Associated) for each individual in the typical N400 time window (300–500 ms).

##### Correlation analysis

N400 effect estimates were computed for each individual as described above for each quadrant of electrodes tested in the previous ANOVAs (Right/Frontal, Left/Frontal, Right/Posterior, Left/Posterior). These quadrants were chosen because the ANOVA above revealed differences in the topographic distribution of the effects of congruence and association. The two posterior quadrants include electrode sites for which the N400 is typically maximal (see Swaab et al., 2011 for a review). The results of correlation tests revealed significant correlations between (1) Listening Span and the effect of congruence in the Right/Frontal quadrant, and (2) Listening Span and the effects of congruence and association in the Left/Posterior quadrant. Additionally, the effect of congruence was correlated with the effect of association in the Left/Posterior quadrant, such that larger effects of congruence were linked to larger effects of association. These results are reported in Table 4.

**Table 4:** **Results of the correlation analyses**.

Variable	1	2	3	4
**RIGHT/FRONTAL QUADRANT**				
1. Congruence effect				
2. Association effect	0.055			
3. Stroop interference	0.037	0.099		
4. Listening span	−0.486*	0.001	0.094	
**LEFT/FRONTAL QUADRANT**				
1. Congruence effect				
2. Association effect	0.223			
3. Stroop interference	−0.125	0.05		
4. Listening span	−0.283	−0.044	0.094	
**RIGHT/POSTERIOR QUADRANT**				
1. Congruence effect				
2. Association effect	−0.225			
3. Stroop interference	0.262	0.035		
4. Listening span	−0.032	0.159	0.094	
**LEFT/POSTERIOR QUADRANT**				
1. Congruence effect				
2. Association effect	0.455*			
3. Stroop interference	−0.132	0.131		
4. Listening span	0.516**	0.622**	0.094	

*Correlations among ERP effects of congruence and association and behavioral measures of Stroop Interference and Listening Span were tested for each quadrant, separately*.

*p < 0.05, **p < 0.01

##### Multiple regression

**Figure 2:**
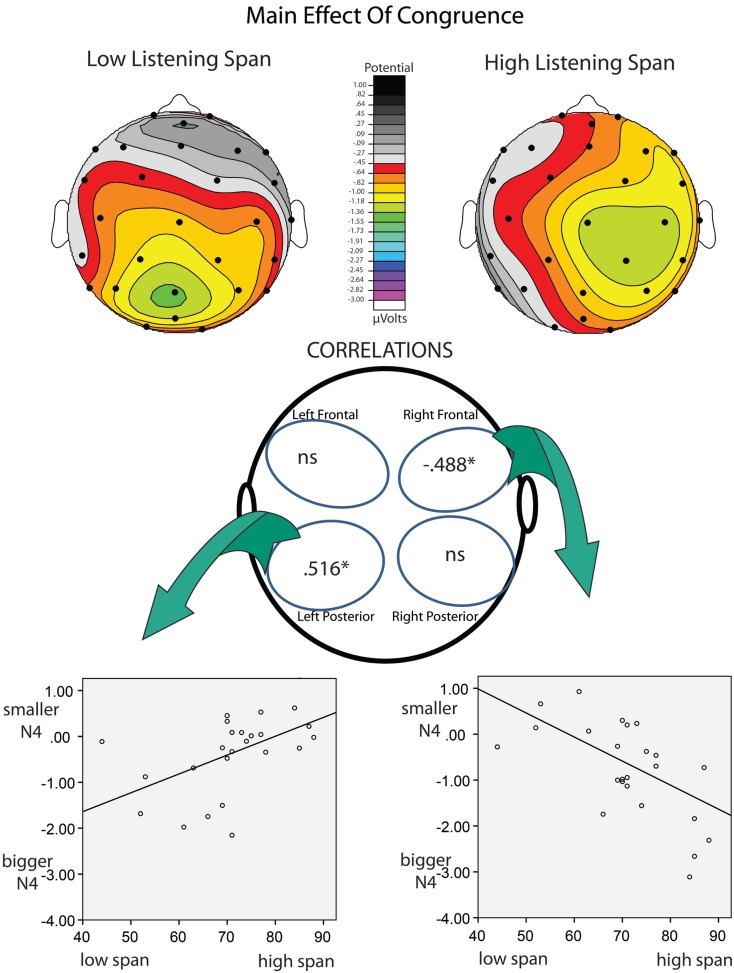
**Correlations of listening span and congruence**. At top are topographic maps showing the scalp distribution of the N400 effect of Discourse Congruence (Incongruent – Congruent) for High-Span participants (left) and Low-Span participants (right). Correlations are shown at bottom. It is important to note that larger N400 effects are reflected by more negative values.

**Figure 3:**
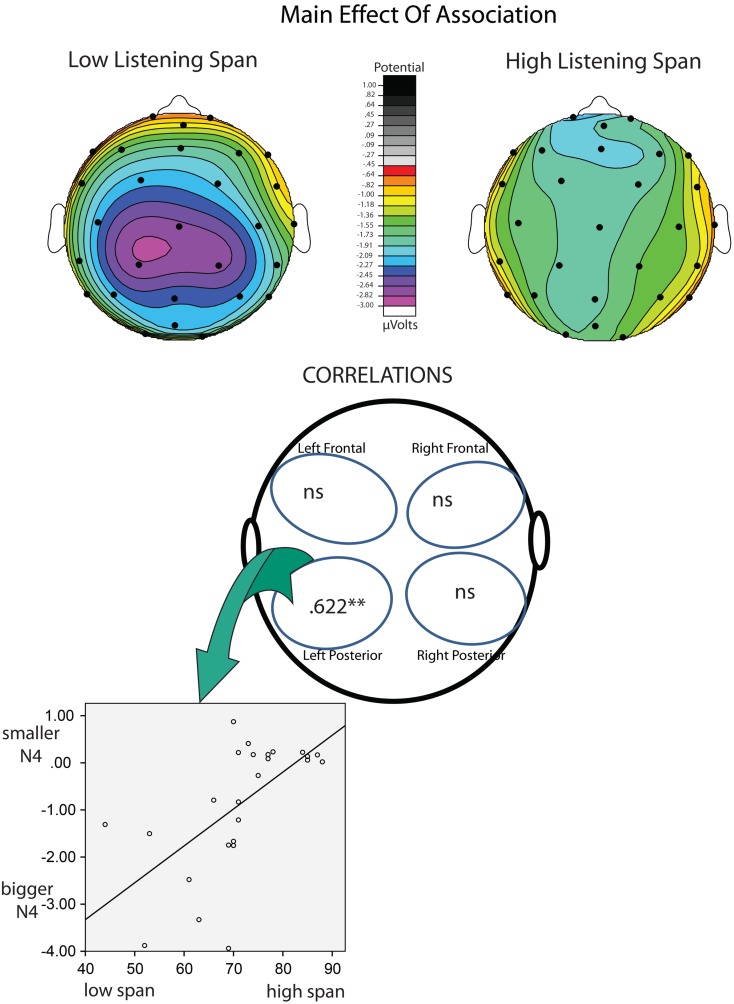
**Correlations of listening span and association**. At top are topographic maps showing the scalp distribution of the N400 effect of Lexical Association (Unassociated – Associated) for High-Span participants (left) and Low-Span participants (right). Correlations are shown at bottom. It is important to note that larger N400 effects are reflected by more negative values.

Listening Span and Stroop Interference were entered into a multiple regression analysis as predictors of N400 effects of congruence and association. This approach allowed us to assess the unique contribution of these two measures in predicting the size of the N400 effects. Stroop Interference did not emerge as a significant predictor of any ERP effects tested in any quadrant. In contrast, Listening Span significantly predicted (1) the effect of congruence in the Right/Frontal [*r* = 0.493 (*r^2^* = 0.243; *p* < 0.05)] and Left/Posterior quadrants [*r* = 0.547 (*r^2^* = 0.299; *p* < 0.01)], and (2) the effect of association in the Left/Posterior quadrants [*r* = 0.626 (*r^2^* = 0.392; *p* < 0.001; see Tables 5 and 6)]. Figures 2 and 3 depict the relations between effects of Listening Span and ERP effects of Discourse Congruence and Association. For illustrative purposes only, participants were divided into High Listening Span (*n* = 12) and Low Listening Span (*n* = 12) groups, excluding participants at the median in order to display differences in topographic distribution as a function of Span.

**Table 5:** **Multiple regression analysis of discourse congruence effect size (two predictors) showing unstandardized (*b*) and standardized (β) partial coefficients, and probability levels (*p*)**.

Predictor	*b*	β	*p*
**LEFT/FRONTAL**			
Constant	1.718		
Listening span	−0.03	−0.274	0.194
Stroop interference	−0.002	−0.099	0.632
**RIGHT/FRONTAL**			
Constant	3.055		
Listening span	−0.053	−0.494	0.015*
Stroop interference	0.002	0.083	0.66
**LEFT/POSTERIOR**			
Constant	−3.236		
Listening span	0.042	0.533	0.007**
Stroop interference	−0.003	−0.183	0.319
**RIGHT/POSTERIOR**			
Constant	−0.776		
Listening span	−0.007	−0.057	0.785
Stroop interference	0.006	0.268	0.208

**Table 6:** **Multiple regression analysis of lexical association effect size (two predictors) showing unstandardized (*b*) and standardized (β) partial coefficients, and probability levels (*p*)**.

Predictor	*b*	β	*p*
**LEFT/FRONTAL**			
Constant	−0.935		
Listening span	−0.006	−0.049	0.819
Stroop interference	0.001	0.055	0.8
**RIGHT/FRONTAL**			
Constant	−1.285		
Listening span	−0.001	−0.009	0.967
Stroop interference	0.002	0.1	0.645
**LEFT/POSTERIOR**			
Constant	−6.49		
Listening span	0.077	0.615	0.001***
Stroop interference	0.002	0.073	0.666
**RIGHT/POSTERIOR**			
Constant	−2.899		
Listening span	0.02	0.157	0.466
Stroop interference	0	0.02	0.925

## Discussion

The goal of this study was to investigate if and how individual differences in cognitive control and WM span affect the processing of discourse-level congruence and word-level meaning relations. A multiple regression approach was used in order to assess the unique influence of these measures on the amplitude of individual N400 effects of congruence and association. Across all participants, significant effects of lexical association and discourse congruence were observed. However, the effect of congruence was marginal in the Left/Frontal quadrant, and was only significant at some sites in the Left/Posterior quadrant. This was surprising, given that discourse congruence effects have previously been found to be robust in comparison to effects of lexical association in this paradigm, as noted in the introduction (Camblin et al., 2007; Boudewyn et al., 2012a). This difference in the relative size of the effects underscores the variability amongst individuals in sensitivity to different aspects of language context. Indeed, as the regression analysis revealed, the different topographic distribution of the effects of congruence varied as a function of Listening Span. These topographic differences could have contributed to the reduced size of the congruence effect in this study compared to in previous work. In addition, Listening Span, but not Stroop Interference, significantly predicted the size of the N400 effects of association in the Left/Posterior quadrant, with higher-span participants showing smaller effects of association than lower-span participants. Interestingly, Listening Span also predicted the size of the N400 effect of discourse congruence, but the direction of the effect varied by topographic distribution: in the Left/Posterior quadrant, higher-span participants had smaller effects of congruence than did lower-span participants, while in the Right/Frontal quadrant, higher-span participants had larger effects of congruence. This pattern is depicted in Figure 3, and suggests that there is a difference in the neural substrates recruited by high compared to low listening span individuals during discourse processing.

The links between Listening Span and the ERP effects of congruence and association were consistent with our predictions, suggesting that higher WM span individuals are less sensitive to the presence of word-level associations within discourse context than lower WM span individuals. Local, word-level context may be more influential, or salient, for individuals with lower WM spans, potentially because lower WM span participants may have less of the discourse context actively maintained and available in WM. If so, then it would follow that the local context immediately preceding the critical target words (i.e., the associated primes) would feature prominently in the portion of context that is active. The pattern of results for the discourse congruence manipulation was complex: higher WM span was not invariably linked with larger ERP effects of congruence, but rather was linked with right *frontal* ERP effects of congruence. In contrast, lower WM span was predictive of left posterior congruence effects. Previous work has also suggested a link between WM span and the use of discourse context (Cantor and Engle, 1993), as well as sentence context (Van Petten et al., 1997; Nakano et al., 2010). Specifically, as mentioned in the introduction, behavioral work has shown that higher WM individuals may be better able than lower-capacity individuals to construct coherent discourse representations (Cantor and Engle, 1993). In addition, ERP work has indicated that high WM individuals are more sensitive to sentence-level congruence than low WM individuals (Van Petten et al., 1997). The results of the current study are novel in showing not only that N400 effects of discourse congruence are linked to WM span, but that WM span predicts topographic differences in the distribution of the effects.

It is interesting that the topography of the N400 effects of congruence, but not of association, varied with listening span. As discussed above, recently encountered prime words may be more salient to low-span individuals than to high-span individuals, resulting in the link between low-span and larger association effects over more classic, central posterior electrode sites. In contrast, congruence effects place greater demands on maintenance and integration over time, and the non-canonical, frontal distribution of this effect for the high listening span individuals is particularly interesting given that frontal negativities in this time window have often been associated with memory manipulations, especially those associated with familiarity (Rugg and Curran, 2007; but see Voss and Federmeier, 2011) and controlled maintenance of verbal WM (Kiss et al., 2007). Although we cannot infer the underlying neural generators from the scalp topography of ERP effects, differences in scalp distribution *do* indicate differences in the configuration and/or number of neural generators producing the effect. Studies using intracranial recordings have implicated the left, and possibly the right, temporal lobes as likely generators of the scalp-recorded N400 effect (Nobre et al., 1994; Nobre and McCarthy, 1995). However, other studies have suggested a possible generator in left inferior frontal cortex (i.e., DLPFC), using fMRI and MEG (Halgren et al., 2002; Hagoort et al., 2004). Therefore, it is an interesting possibility that the more frontal N400 effect of discourse congruence than was seen in the higher listening span participants in the current study is driven by the recruitment or increased engagement of prefrontal cortex during controlled maintenance and use of discourse-level context.

It is also interesting that the higher-span participants showed larger effects of discourse congruence over *right* frontal electrode sites than did lower-span participants. Again, we cannot conclude based on the ERP data in this study alone that this was caused by a right hemisphere (RH) generator. However, there is a rich literature that implicates the RH in discourse processing (e.g., St. George et al., 1999; Xu et al., 2005; see also Johns et al., 2008, for a review). Specifically, there is evidence for increased engagement of the RH during comprehension as complexity of the language input increases, with RH activation being maximal at the discourse-level of processing (e.g., Xu et al., 2005). It is possible that, in the current study, increased sensitivity to the discourse-level meaning representation for higher WM capacity individuals led to increased RH involvement, which could have contributed to the relation among WM span, discourse congruence effects, and topographic distribution that we observed.

The lack of a link between our measure of control, Stroop Interference, and the ERP effects of either discourse congruence or lexical association was unexpected. We predicted that Stroop Interference would be predictive of increased lexical association effects, which we have previously found to be the case during the comprehension of short sentences (Boudewyn et al., 2012a). In Boudewyn et al., participants listened to simple spoken sentences that averaged only 11 words per sentence, including the prime and target pairs. Therefore, the context prior to the critical words was minimal and unlikely to tax WM; indeed, WM did not predict the N400 effect of lexical association in that study. Instead, Stroop Interference significantly predicted the N400 effect of lexical association, with higher-control individuals showing smaller association effects than lower-control individuals. This was interpreted as evidence that higher-control (better suppression ability) individuals were more effective than lower-control individuals at suppressing the word-level meaning relation, which represents information that is somewhat irrelevant in a sentence processing context (Boudewyn et al., 2012a). Previous studies of word priming effects have also suggested that small or absent priming effects may be attributed to suppression of the semantic relation rather than to an absence of semantic activation (Mari-Beffa et al., 2000), and that lexical associates of words in sentence contexts may be activated but then inhibited, when the task demands are to understand the sentence as a whole (Norris et al., 2006). In the current study, however, Stroop Interference was not significantly related to the effects of local lexical association.

In our view, there are at least two possible interpretations of this result. The first is that, when associated prime-target pairs that are embedded in sentences are additionally embedded within discourse context, sensitivity to the local meaning association is primarily determined by WM rather than by control/suppression ability. There is some reason to think that this may be the case: as discussed previously, cognitive control as measured by the Stroop task in the current study primarily taps into suppression ability and conflict resolution, rather than controlled maintenance of information over time. In contrast, our Listening Span task *does* require the maintenance of context information over time. Therefore, when a larger context is available to the language user, it may be the case that Listening Span accounts for individual differences in sensitivity to both discourse congruence and lexical association better than Stroop Interference. In other words, although sensitivity to the presence of word associations may be largely determined by suppression ability when there is a minimal maintenance load (simple sentence context), the added maintenance demands of a larger, more complex discourse context may result in context representations that vary in the prominence of the prime word, as a function of WM span. Individuals with high WM span are likely to maintain representations in which a greater amount of context information is active and available; thus, the local prime words would feature less prominently. Individuals with low WM span may have less of the preceding context available in memory, leading the local prime words to occupy a more prominent and salient position in memory when the target word is then encountered. Under these circumstances, WM span may trump suppression ability as the dominating factor influencing sensitivity to local associations during the processing of the incoming target words.

We cannot completely rule out the possibility that suppression ability does play a role in determining the size of individual N400 effects of lexical association, even for words in discourse context, but that it simply failed to emerge as significant in the current study. This could be a result of insufficient power, but we do not think this to be the case because there was sufficient power to detect significant relations among Listening Span and our ERP effects of interest. Another possible explanation is that participant strategy influenced our measure of Stroop Interference: on average, participants in the current study had slower reaction times on all trial types in the Stroop Interference task than those reported by Boudewyn et al., 2012a[644.21 and 697.75 ms for congruent (C) and RI words in the current study, compared to 604.95 and 677.68 ms in Boudewyn et al., 2012a]. It is possible that participants in the current study adopted a more conservative strategy on this task, taking longer to respond and potentially making this measure less sensitive to raw suppression ability, as a result. It is important to keep in mind, however, that the lack of significant relations among Stroop Interference and ERP effects of lexical association and discourse congruence does not imply that control processes, and specifically suppression ability, are unimportant in discourse comprehension. Instead, it suggests that the maintenance of context information over time may be the aspect of controlled processing that best determines sensitivity to multiple levels of meaning during discourse comprehension.

## Conclusion

The results of this study show that listeners vary in sensitivity to local lexical associations embedded within discourse context as a function of WM span. High-span individuals are less sensitive to the presence of associatively related prime words in context. WM span was also related to differences in the topographic distribution of effects of discourse congruence: N400 effects of discourse congruence were maximal for high-span participants at right frontal electrode sites, whereas congruence effects were maximal for low-span participants at left posterior electrode sites. This topographic difference in the distribution of the effects can only be explained by differences in the configuration of the underlying neural generators of the N400 effects of congruence, as a function of WM capacity. This pattern of results suggests additional, or at least distinct, processing on the part of higher-span individuals when integrating incoming input with the current discourse representation.

## Conflict of Interest Statement

The authors declare that the research was conducted in the absence of any commercial or financial relationships that could be construed as a potential conflict of interest.

## Footnotes

^1^ The duration of this pause was set to 1 s in order to match the average of the naturally produced pauses between sentences one and two.
